# Sexual identity, child maltreatment, mental health, and substance use among emerging adults aged 18 to 23 years

**DOI:** 10.17269/s41997-024-00992-5

**Published:** 2025-02-28

**Authors:** Tracie O. Afifi, Ana Osorio, Janique Fortier, Ashley Stewart-Tufescu, Tamara L. Taillieu, Julie-Anne McCarthy

**Affiliations:** 1 Department of Community Health Sciences, University of Manitoba, Winnipeg, MB Canada; 2 Department of Psychiatry, University of Manitoba, Winnipeg, MB Canada; 3 Faculty of Social Work, University of Manitoba, Winnipeg, MB Canada

**Keywords:** Sexual identity, Sexual orientation, Child maltreatment, Depression, Anxiety, Cannabis, Alcohol, Substance use, Identité sexuelle, Orientation sexuelle, Maltraitance des enfants, Dépression, Anxiété, Cannabis, Alcool, Consommation de substances

## Abstract

**Objectives:**

Although past studies have identified sex differences in child maltreatment experiences and poor mental and physical health‒related outcomes, more research is needed to understand child maltreatment among sexual minorities (i.e., those who identify as other than heterosexual) and how child maltreatment and sexual identity are related to depression, anxiety, and at-risk alcohol and cannabis use among emerging adults.

**Methods:**

Data were drawn from the longitudinal Well-Being and Experiences (WE) Study collected from 2017 (14 to 17 years) to 2022 (18 to 23 years) from Manitoba, Canada (*n* = 584). Descriptive statistics and logistic regression models were computed.

**Results:**

Compared to heterosexual or straight sexual identity: homosexual, gay or lesbian; bisexual; and different or other identity were associated with an increased likelihood of experiencing child maltreatment, with the most robust relationships for bisexual identity and all child maltreatment outcomes. Indicating “I don’t know” for sexual identity compared to heterosexual identity was associated with 7.45 increased odds of exposure to intimate partner violence in adjusted models. Bisexual identity compared to heterosexual identity had the most robust association, with increased odds of depression, anxiety, at-risk alcohol use, and at-risk cannabis use. Findings provide some evidence to suggest that trends may be worse for some mental health and substance use outcomes among sexual minorities who also experience child maltreatment.

**Conclusion:**

Preventing child maltreatment among all children, including youth identifying as other than heterosexual, is a public health priority. Such efforts will work towards optimizing mental health and reducing substance use in early adulthood.

## Résumé

**Objectifs:**

Des études antérieures ont constaté des différences entre les sexes dans les expériences de maltraitance des enfants et les problèmes de santé mentale et physique qui en résultent, mais il faut pousser la recherche pour comprendre la maltraitance des enfants chez les minorités sexuelles (c.-à-d. les personnes qui s’identifient autrement que comme étant hétérosexuelles) et les liens entre la maltraitance des enfants et l’identité sexuelle, d’une part, et la dépression, l’anxiété et la consommation à risque d’alcool et de cannabis chez les adultes en émergence.

**Méthode:**

Les données proviennent de l’étude longitudinale Well-Being and Experiences (WE); elles ont été recueillies entre 2017 (14 à 17 ans) et 2022 (18 à 23 ans) au Manitoba (Canada) (*n* = 584). Des statistiques descriptives et des modèles de régression logistique ont été employés.

**Résultats:**

Comparativement à l’identité hétérosexuelle (hétéro): l’identité homosexuelle, gaie ou lesbienne, l’identité bisexuelle et les identités différentes ou autres étaient associées à une probabilité accrue d’avoir connu la maltraitance durant l’enfance; les relations les plus robustes ont été observées pour l’identité bisexuelle et pour tous les résultats de maltraitance des enfants. La réponse «Je ne sais pas» à la question sur l’identité sexuelle, comparativement à l’identité hétérosexuelle, était associée à une probabilité d’exposition à la violence entre partenaires intimes accrue de 7,45 dans les modèles ajustés. L’identité bisexuelle, comparativement à l’identité hétérosexuelle, présentait l’association la plus robuste avec les probabilités accrues de dépression, d’anxiété, de consommation d’alcool à risque et de consommation de cannabis à risque. Les constats de l’étude donnent à penser que les tendances peuvent être pires pour certains résultats de santé mentale et de consommation de substances chez les minorités sexuelles ayant aussi connu la maltraitance durant l’enfance.

**Conclusion:**

La prévention de la maltraitance chez tous les enfants, y compris les jeunes s’identifiant autrement que comme étant hétérosexuels, est un besoin prioritaire en santé publique. Une telle démarche contribuera à optimiser la santé mentale et à réduire la consommation de substances au début de l’âge adulte.

## Introduction

Child maltreatment (i.e., child abuse and neglect) remains an important public health problem that can have a negative impact on health, substance use, criminal behaviour, and risky sexual behaviour across the lifespan (Gilbert et al., 2009). Child maltreatment includes experiences of physical abuse, sexual abuse, emotional abuse, emotional neglect, physical neglect, and exposure to intimate partner violence (EIPV). It is estimated that every year, millions of children are abused and neglected worldwide (World Health Organization, 2014; World Health Organization et al., 2006). In Canada, 32% of adults in the general population retrospectively reported having experienced physical abuse, sexual abuse, and/or EIPV in childhood (Afifi et al., 2014). When child maltreatment occurs, the odds of having substance use problems, mental disorders, and thinking about or attempting suicide increase (Afifi et al., 2011, 2012a, 2013, 2014, 2017a, 2020b; Anda et al., 2002; Dube et al., 2002; Felitti et al., 1998; Merrick et al., 2017). Although advances have been made in understanding the impact of child maltreatment, several gaps in knowledge remain, including a lack of understanding of these associations among individuals with diverse sexual identities.

Research has shown that sex differences (i.e., biological attributes and sex assigned as male or female at birth) exist in relation to the prevalence of different child abuse types in Canada, with males (31%) being more likely to experience physical abuse than females (21.3%), and females (14.4%) being more likely to experience sexual abuse than males (5.8%) (Afifi et al., 2014). In addition to sex differences in the prevalence of child abuse types, sex differences are noted in the literature related to relationships between child maltreatment and poor mental and physical health outcomes, including substance use during adolescence (Benedini & Fagan, 2020; Salmon et al., 2022) and into adulthood (Afifi et al., 2012b, 2017b; Gallo et al., 2018; MacMillan et al., 2001; Ruiz & Font, 2020; Scott et al., 2023; Soares et al., 2020).

Although several studies have included sex differences when examining the relationships between child maltreatment and mental health and substance use, fewer studies have examined how sexual orientation or sexual identity may be related to child maltreatment. Sexual orientation refers to emotional, physical, romantic, sexual or spiritual attraction; desire; or affection for another person (GLAAD, n.d.; Pinto et al., 2019). In this study, we use the term sexual identity to refer to how an individual identifies their sexual orientation. Sexual minorities are those who identify as other than heterosexual. Previous research using nationally representative Canadian data of youth aged 15 to 17 years collected in 2019 indicated that 78.6% identified as heterosexual, 0.8% identified as boys attracted to boys, 1.0% identified as girls attracted to girls, 14.7% identified as attracted to more than one gender, 0.6% identified as transgender, and 4.3% identified as not sure about sexual attraction (Kingsbury et al., 2022). Research indicates that adults and youth identifying with a sexual identity other than heterosexual report elevated levels of adverse childhood experiences (ACEs), including child abuse and neglect (Afifi et al., 2012b; Andersen & Blosnich, 2013; Austin et al., 2016; Clements-Nolle et al., 2018; Craig et al., 2020; Friedman et al., 2011; Schnarrs et al., 2019; Tran et al., 2023). For example, women who identify as lesbian, bisexual, or gay have reported high levels of child physical and sexual abuse (Morris & Balsam, 2003). Parental acceptance and rejection theories that may impact parent–child attachment have been used to understand why sexual minority youth born to heterosexual parents may be at-risk for violence (Katz-Wise et al., 2016). For some youth, expressing sexual identity that is other than heterosexual may create negative parental responses that may lead to anxiety for the child, future abuse, or banishment from the home (Katz-Wise et al., 2016). In addition, previous research has indicated that anxiety, depression, suicide attempts, and substance use are elevated among youth and adults who identify as gay, lesbian, or bisexual in comparison to heterosexual (Depa et al., 2022; Krueger et al., 2022; McCabe et al., 2022; Morris & Balsam, 2003), with some studies indicating that bisexual individuals have the most elevated associations (Chan et al., 2020; Charak et al., 2019; Friedman et al., 2011; McCabe et al., 2022).

The current literature on sexual identity and mental health and substance use outcomes remains limited for several reasons. First, as previously mentioned, most studies look at sex differences and not sexual identity differences. Second, research that includes sexual identity often uses undergraduate samples or crowdsourcing (Balsam et al., 2010; Charak et al., 2019; Craig et al., 2017; Jiang et al., 2023). Third, some studies do not use validated measures of child maltreatment, mental disorders, and/or substance use (Andresen et al., 2022; Schnarrs et al., 2019). Fourth, it can be common for studies to only include one type of child maltreatment, such as sexual abuse (McCabe et al., 2022). The inclusion of emotional abuse, emotional neglect, and physical neglect is less common in this literature. While these data on sexual abuse are still important and useful, understanding experiences more widely, including other types of child maltreatment, is needed. Fifth, adjusting for sociodemographic covariates, including sex, age, household income, and ethnicity, is important for understanding the independent relationship between sexual identity and mental health and substance use outcomes. Finally, more research in this area is necessary among youth and emerging adults since studies often use adult-only samples with wide age ranges (Andresen et al., 2022; Friedman et al., 2011; Tran et al., 2022). It is important to better understand the relationships between sexual identity and child maltreatment experiences among youth and emerging adults, and to examine whether child maltreatment histories appear to worsen mental health and substance use outcomes.

To address these knowledge gaps, the aims of the current study were to (1) compute the prevalence of sexual identity in a community sample of young emerging adults; (2) determine the associations between sexual identity and increased likelihood of experiencing child maltreatment, including physical abuse, sexual abuse, emotional abuse, physical neglect, emotional neglect, and EIPV; (3) determine the associations between sexual identity and increased likelihood of depression, anxiety, and at-risk alcohol and cannabis use; and (4) examine whether child maltreatment histories moderate the relationships between sexual identity and depression, anxiety, and at-risk alcohol and cannabis use or whether trends in the relationships between sexual identity and mental disorders and substance use appear to be worse for emerging adults with a child maltreatment history.

## Methods

### Data and sample

Data were drawn from the longitudinal Well-Being and Experiences (WE) Study collected from 2017 to 2022 using individual self-administered computerized surveys in Manitoba, Canada. The overall purpose of the WE Study was to learn more about the health, well-being, and experiences of youth, parents, and families in Manitoba. The intended population for the WE Study was youth aged 14 to 17 years along with a parent or caregiver. Convenience sampling (i.e., referrals and community advertisements; 79%) and random digit dialing (21%) were used to recruit respondents. During baseline data collection, demographic variables, including sex, household income, and ethnicity, were monitored to ensure the sample resembled the province’s general population (Statistics Canada, 2017). For the current study, data were from waves 1, 3, 4, and 5 (*n* = 584, 58% retained from baseline in 2017). No data from wave 2 were required for the current study. Adolescents were 14 to 17 years old at wave 1 in 2017–2018 and were 18 to 23 years old at wave 5. Wave 1 questionnaires were completed in person in private rooms using an online survey at a research facility. Waves 3–5 questionnaires were completed using an emailed link to an online survey. Informed consent was provided at all waves. Ethics approval was obtained from the Health Research Ethics Board at the University of Manitoba. Additional information about the WE Study has been previously published (Afifi et al., 2020a, 2020b; Fortier et al., 2022).

### Measurement

#### Sexual identity

Respondents were asked to indicate their current sexual identity at waves 4 and 5. Response options included (a) heterosexual or straight; (b) homosexual, gay, or lesbian; (c) bisexual; (d) other identity (please specify); (e) I don’t know; and (f) no response. Answers at wave 5 (in 2022) were used in this study to measure sexual identity. For individuals who did not participate in wave 5, information on sexual identity provided in wave 4 was used. Thirty-two of the respondents specified their identities (verbatim), which included asexual, asexual and aromantic, pansexual, pansexual and polyamorous, pansexual/demisexual, queer/questioning, queer/asexual spectrum/bisexual or pansexual (uncertain), heterosexual-omniromantic, unidentified, and unlabeled. These identities could not be examined individually due to low frequency and were therefore combined as *other identity*.

#### Child maltreatment

Childhood physical abuse, sexual abuse, emotional abuse, physical neglect, and emotional neglect were measured using the Childhood Trauma Questionnaire (CTQ), which included five items for each type of abuse or neglect (Bernstein & Fink, 1998). EIPV was assessed using an adapted item from the Childhood Experiences of Violence Questionnaire (CEVQ), asking the respondents whether, before age 16 years, they heard a parent, step-parent, or guardian hit each other or another adult in their home (Walsh et al., 2008). Complete child maltreatment information was collected in waves 3, 4, and 5 when the respondent was 18 years old. Any child maltreatment was coded as having experienced one or more of the above types of abuse and/or neglect.

#### Depression and anxiety

The 9-item Patient Health Questionnaire (PHQ-9) was used to assess depression symptoms at waves 3, 4, and 5. Anxiety symptoms were assessed using the 7-item General Anxiety Disorder (GAD-7). For both instruments, respondents rated how bothered they had been by certain problems over the past 2 weeks based on a 4-point scale. A probable diagnosis of depression or anxiety being clinically significant at any of the three waves was coded with a summed score of 10 or more (Kroenke et al., 2010; Manea et al., 2015).

#### At-risk alcohol use and at-risk cannabis use

At-risk alcohol use was assessed using the Alcohol Use Disorders Identification Test (AUDIT) at waves 3, 4, and 5. At-risk cannabis use was assessed using the Cannabis Use Disorder Identification Test–Revised (CUDIT-R) at waves 3, 4, and 5. The AUDIT is a 10-item instrument psychometrically validated that assesses past-year alcohol use, with most questions rated on a 5-point scale (Babor et al., 2001; Saunders et al., 1993). The CUDIT-R is an eight-item revised and psychometrically validated version of the original CUDIT instrument that assesses past 6-month cannabis use, with the majority of questions also rated on a 5-point scale (Adamson et al., 2010). While earlier work suggests a score of eight or more to assess at-risk alcohol use (Babor et al., 2001; Saunders et al., 1993), a number of recent studies testing AUDIT psychometrics among younger populations of adolescents and/or young adults (primarily college or university students) recommend using a lower cut-off score (Cortés-Tomás et al., 2016; Coulton et al., 2019; Kokotailo et al., 2004; Liskola et al., 2018; Reichenheim et al., 2021). Given the age range in our sample, we decided to lower the cut-off score to six or more to assess at-risk alcohol use, as has been used in a previous study of university students in the United States (Kokotailo et al., 2004). Similarly, a cut-off score of six or more was used to assess at-risk cannabis use, based on the recommendation by Schultz et al. (2019).

#### Covariates

Sex at birth (i.e., male or female), age in years, youth ethnicity (i.e., white only, white and another ethnicity, or other/multi-ethnicity), and household income reported by parents at wave 1 (i.e., $49,999 or less, $50,000 to $99,999, $100,000 to $149,999, and $150,000 or more) were included as covariates in adjusted models.

### Statistical analysis

First, descriptive statistics were conducted. Next, logistic regression models were run between sexual identity and (a) each child maltreatment type, (b) depression, (c) anxiety, (d) at-risk alcohol use, and (e) at-risk cannabis use. Models were first run unadjusted and then adjusted for sex, age, ethnicity, and household income. Finally, multiplicative interaction effect models were computed to determine whether child maltreatment moderated (i.e., worsened) the relationships between sexual identity and (a) depression, (b) anxiety, (c) at-risk alcohol use, and (d) at-risk cannabis use. As a post hoc examination, logistic regressions were conducted stratified by child maltreatment (i.e., those with and without a child maltreatment history) to examine whether trends appear worse among those with a child maltreatment history. Three levels of *p* values are provided for the logistic regression models at * *p* ≤ 0.05; ** *p* ≤ 0.01; *** *p* ≤ 0.001 so that one can interpret statistical significance at more conservative levels if desired.

## Results

Among the sample, 70.6% identified as heterosexual; 2.6% as homosexual, gay, or lesbian; 19.5% as bisexual; 3.9% as other identity; and 3.4% as indicating “I don’t know”. Table 1 presents the prevalence of sex, household income, and age by sexual identity.

**Table 1:** Sociodemographic variables by sexual identity among emerging adults in Manitoba, Canada

	Total sample	Heterosexual or straight	Other identity
Sociodemographic	(%)	(%)	(%)
Biological sex (from wave 1)			
Male	(43.89)	(51.34)	(25.88)
Female	(56.11)	(48.66)	(74.12)
Household income (parent reported at wave 1)			
$49,999 or less	(15.41)	(12.72)	(21.82)
$50,000 to $99,999	(38.17)	(37.91)	(38.79)
$100,000 to $149,999	(23.66)	(26.46)	(16.97)
$150,000 or more	(22.76)	(22.90)	(22.42)
Age (at wave 5)			
18	(11.20)	(10.14)	(11.63)
19	(26.72)	(21.62)	(28.81)
20	(25.93)	(29.73)	(24.38)
21	(22.59)	(23.65)	(22.16)
22‒23	(13.56)	(14.86)	(13.02)

Table 2 presents findings of the relationships for sexual identity and odds of experiencing physical abuse, sexual abuse, emotional abuse, physical neglect, and emotional neglect. Bisexual identity compared to heterosexual identity was associated with an increased likelihood of physical abuse (adjusted odds ratio (AOR) = 2.39, 95% confidence interval (CI) = 1.18–4.82 in the adjusted model), sexual abuse (AOR = 3.66, 95% CI = 2.11–6.32), emotional abuse (AOR = 3.35, 95% CI = 1.97–5.68), physical neglect (AOR = 3.60, 95% CI = 2.02–6.44), and emotional neglect (AOR = 2.89, 95% CI = 1.56–5.36). Other identity, compared to heterosexual identity, was associated with increased odds of experiencing physical neglect (AOR = 3.36, 95% CI = 1.14–9.89). Some type II errors may be indicated in non-significant models with moderate effect sizes and large confidence intervals. As a post hoc sensitivity analysis due to limited statistical power, we dichotomized sexual identity as heterosexual versus all other identities and found that having sexual identities other than heterosexual was associated with an increased likelihood of all five child maltreatment types in the adjusted models (AORs ranging from 1.98 to 3.33).

**Table 2:** Associations between sexual identity and child maltreatment among emerging adults in Manitoba, Canada

	Physical abuse		Sexual abuse		Emotional abuse		Physical neglect		Emotional neglect	
	OR (95% CI)	AOR (95% CI)	OR (95% CI)	AOR (95% CI)	OR (95% CI)	AOR (95% CI)	OR (95% CI)	AOR (95% CI)	OR (95% CI)	AOR (95% CI)
Sexual identity (reference group: heterosexual or straight)										
Homosexual, gay, or lesbian	1.96	1.26	2.35	1.93	3.28*	2.27	3.05	3.08	4.00*	3.66
	(0.42–9.14)	(0.15–10.60)	(0.62–8.85)	(0.39–9.57)	(1.06–10.12)	(0.56–9.20)	(0.92–10.13)	(0.76–12.46)	(1.19–13.40)	(0.93–14.45)
Bisexual	2.90***	2.39*	4.42***	3.66***	4.02***	3.35***	3.56***	3.60***	2.98***	2.89***
	(1.59–5.30)	(1.18–4.82)	(2.68–7.29)	(2.11–6.32)	(2.51–6.43)	(1.97–5.68)	(2.15–5.90)	(2.02–6.44)	(1.70–5.23)	(1.56–5.36)
Other identity	1.17	1.11	1.24	1.18	1.73	1.73	3.05*	3.36*	2.22	2.38
	(0.26–5.26)	(0.23–5.22)	(0.35–4.34)	(0.33–4.28)	(0.62–4.88)	(0.59–5.11)	(1.13–8.26)	(1.14–9.89)	(0.71–6.92)	(0.74–7.63)
I don’t know	1.47	1.59	1.47	1.52	1.57	1.74	2.03	2.2	2.67	2.62
	(0.32–6.68)	(0.33–7.64)	(0.41–5.23)	(0.41–5.69)	(0.50–4.90)	(0.53–5.65)	(0.65–6.39)	(0.66–7.35)	(0.84–8.46)	(0.79–8.65)
Sensitivity analysis (reference group: heterosexual or straight)										
Homosexual, gay, or lesbian, bisexual, other, I don’t know	2.38***	1.98*	3.29***	2.82***	3.25***	2.78***	3.25***	3.33***	2.91***	2.84***
	(1.37–4.14)	(1.05–3.72)	(2.08–5.21)	(1.71–4.63)	(2.13–4.96)	(1.74–4.45)	(2.06–5.12)	(2.00–5.55)	(1.76–4.81)	(1.65–4.89)

*OR*, odds ratio; *CI*, confidence interval; *AOR*, adjusted odds ratio. AOR adjusted for biological sex, age, ethnicity, and household income

^***^p < 0.001; **p < 0.01; *p < 0.05

Table 3 presents the findings for sexual identity and odds of experiencing EIPV. Indicating “I don’t know” for sexual identity compared to heterosexual was associated with increased odds for EIPV (AOR = 7.45, 95% CI = 1.72–32.34).

**Table 3:** Associations between sexual identity and child maltreatment among emerging adults in Manitoba, Canada

	Exposure to verbal intimate partner violence	
	OR (95% CI)	AOR (95% CI)
Sexual identity (reference group: heterosexual or straight)		
Homosexual, gay, or lesbian	Omitted	Omitted
Bisexual	3.12**	2.08
	(1.33–7.33)	(0.77–5.63)
Other identity	1.40	1.38
	(0.17–11.18)	(0.16–11.70)
I don’t know	5.50*	7.45**
	(1.42–21.23)	(1.72–32.34)
Sensitivity analysis (reference group: heterosexual or straight)		
Homosexual, gay, or lesbian, bisexual, other, I don’t know	2.83**	2.25
	(1.30–6.17)	(0.94–5.41)

*OR*, odds ratio; *CI*, confidence interval; *AOR*, adjusted odds ratio. AOR adjusted for biological sex, age, ethnicity, and household income

^***^p < 0.001; **p < 0.01; *p < 0.05

Table 4 presents the findings of the relationships between sexual identity and mental health and substance use outcomes. Sexual identity of homosexual, gay, or lesbian compared to heterosexual identity was associated with an increased likelihood of depression (AOR = 12.05, 95% CI = 1.50–96.67) and at-risk cannabis use (AOR = 4.67, 95% CI = 1.34–16.20). Bisexual identity, compared to heterosexual identity, was associated with an increased likelihood of depression (AOR = 5.33, 95% CI = 2.81–10.12), anxiety (AOR = 2.07, 95% CI = 1.25–3.43), at-risk alcohol use (AOR = 2.05, 95% CI = 1.25–3.34), and at-risk cannabis use (AOR = 2.67, 95% CI = 1.62–4.41). Other identity, compared to heterosexual identity, was associated with an increased likelihood of depression (AOR = 4.32, 95% CI = 1.22–15.24). Notably, several of these models indicated moderate effect sizes with wide confidence intervals that were not statistically significant, which indicates some underpowered models and a possibility of a type II error.

**Table 4:** Associations between sexual identity and mental health and substance use among emerging adults in Manitoba, Canada

	Depression		Anxiety		CUDIT		AUDIT	
	OR (95% CI)	AOR (95% CI)	OR (95% CI)	AOR (95% CI)	OR (95% CI)	AOR (95% CI)	OR (95% CI)	AOR (95% CI)
Sexual identity (ref: heterosexual or straight)								
Homosexual, gay, or lesbian	13.15*	12.05*	2.34	1.76	3.10*	4.67*	0.80	0.69
	(1.71–100.97)	(1.50–96.67)	(0.77–7.11)	(0.50–6.17)	(1.08–8.92)	(1.34–16.20)	(0.26–2.48)	(0.19–2.48)
Bisexual	6.31***	5.33***	2.87***	2.07**	2.62***	2.67***	2.05**	2.05**
	(3.47–11.45)	(2.81–10.12)	(1.82–4.55)	(1.25–3.43)	(1.67–4.10)	(1.62–4.41)	(1.32–3.19)	(1.25–3.34)
Other identity	4.23*	4.32*	2.02	2.09	1.55	1.97	1.04	1.25
	(1.40–12.72)	(1.22–15.24)	(0.85–4.78)	(0.80–5.46)	(0.64–3.79)	(0.76–5.10)	(0.42–2.58)	(0.48–3.26)
I don’t know	3.52*	3.00	2.81*	2.49	1.50	1.43	0.93	0.82
	(1.15–10.80)	(0.95–9.47)	(1.05–7.56)	(0.89–6.97)	(0.59–3.84)	(0.54–3.83)	(0.36–2.36)	(0.30–2.26)

*OR*, odds ratio; *CI*, confidence interval; *AOR*, adjusted odds ratio. AOR adjusted for biological sex, age, ethnicity, and household income

^***^p < 0.001; **p < 0.01; *p < 0.05

Moderation models were computed and assessed at *p* value of 0.1 as recommended to reduce a type II error when sample sizes are small (Fairchild & MacKinnon, 2009). None of the models reached statistical significance and, therefore, post hoc logistic regression models were computed stratified by child maltreatment histories to examine overall trends. Table 5 presents the findings for the relationships between sexual identity and depression, anxiety, at-risk alcohol use, and at-risk cannabis use stratified by those with and without a child maltreatment history. Although not statistically compared due to stratified models, trends suggest that sexual identity is related to poor mental health and at-risk substance use for those with and without a child maltreatment history, with some larger effects sizes for homosexual, gay, or lesbian compared to heterosexual individuals for anxiety, at-risk alcohol, and at-risk cannabis use; bisexual compared to heterosexual individuals for depression and anxiety; and those indicating “I don’t know” for sexual identity compared to heterosexuals for at-risk alcohol use.

**Table 5:** Associations between sexual identity and mental health and substance use stratified by child maltreatment

	Depression		Anxiety		At-risk cannabis		At-risk alcohol	
	OR (95% CI)		OR (95% CI)		OR (95% CI)		OR (95% CI)	
	Any child maltreatment							
Sexual identity (ref: heterosexual or straight)	No	Yes	No	Yes	No	Yes	No	Yes
Homosexual, gay, or lesbian	7.78	Omitted	1.45	3.06	3.45	3.67	0.27	1.65
	(0.92–65.57)		(0.32–6.61)	(0.35–27.02)	(0.75–15.82)	(0.68–19.73)	(0.03–2.32)	(0.35–7.70)
Bisexual	2.98**	8.16***	1.50	2.28*	2.43*	1.99*	2.06	1.85*
	(1.36–6.53)	(2.77–24.05)	(0.71–3.16)	(1.15–4.51)	(1.16–5.10)	(1.07–3.69)	(0.98–4.32)	(1.01–3.39)
Other identity	11.67*	1.3	2.31	1.07	2.58	0.84	1.67	0.71
	(1.46–93.50)	(0.33–5.18)	(0.69–7.81)	(0.30–3.86)	(0.72–9.22)	(0.23–3.03)	(0.44–6.37)	(0.20–2.55)
I don’t know	2.92	Omitted	2.25	Omitted	1.62	1.47	0.67	1.24
	(0.87–9.72)		(0.73–6.91)		(0.51–5.12)	(0.28–7.60)	(0.20–2.28)	(0.24–6.38)

*OR*, odds ratio. Omitted because predicts success perfectly. ***p < 0.001; **p < 0.01; *p < 0.05

## Discussion

Novel findings from this research include furthering our understanding of how several sexual identities are related to child maltreatment types, depression, anxiety, at-risk alcohol use, and at-risk cannabis use among a community sample of emerging adults. Findings indicate that compared to identifying as heterosexual, sexual identity of homosexual, gay, or lesbian; bisexual; and other identity were associated with an increased likelihood of having a child maltreatment history, along with an increased likelihood of many poor mental health and substance use outcomes. Notably, more significant models were found for depression and anxiety and fewer for at-risk cannabis and at-risk alcohol. When looking at the analysis stratified by those with and without a child maltreatment history, different trends were noted, suggesting that the association between sexual identity and poor mental health and substance use outcomes may be worsened in some cases when sexual minority individuals also experience child maltreatment. Findings from the current study affirm previous studies that indicate that individuals who identify as other than heterosexual are at an increased likelihood of experiencing childhood violence (Andersen & Blosnich, 2013; Austin et al., 2016, 2022; Charak et al., 2019; Clements-Nolle et al., 2018; Friedman et al., 2011; Jiang et al., 2023; Schnarrs et al., 2019; Tran et al., 2022). Our findings advance this work with further detailed analyses that include several sexual identities and several child maltreatment types.

From a human rights perspective, children and youth have the right to live free from all forms of violence (Whalen, 2022). Policies are needed to protect children when it is noted that some children and youth may have a greater likelihood of violence exposure. Promoting and maintaining safe environments for all children is essential to ensure healthy development and to reduce mental disorders and at-risk substance use. This may be especially necessary for children and youth who identify with a sexual identity other than heterosexual. From a societal perspective, it is important to invest in public education and resources that normalize and promote acceptance of all sexual identities and remove any tolerance for violence against children and youth. Parenting programs that educate caregivers about children’s development, including sexual and gender identity, orientation, and expression, may prevent and reduce childhood violence. It is also important for clinicians to be aware of the relationships between sexual identity, child maltreatment, depression, anxiety, and at-risk substance use when an individual accesses care in an effort to improve mental health as individuals move from adolescence into adulthood.

### Limitations

Findings from the current study should be interpreted while considering the limitations of the research. First, although the sample was similar to the general population of Manitoba based on sex and household income at the first baseline data collection (Statistics Canada, 2017), because the data collection methods were non-random and due to attrition since baseline, we cannot refer to this as a representative sample. Second, only one geographical area in Canada was included in the study. Third, it is possible that sexual minorities may underreport identities, resulting in some misclassification. Fourth, we did not have adequate power to examine gender identity. This would be important to investigate in future work of emerging adults. Finally, future work with a larger sample size should include moderation analyses to confirm whether a child maltreatment history significantly modifies or worsens the relationship between sexual identity and depression, anxiety, at-risk alcohol use, and at-risk cannabis use.

## Conclusion

Although there are notable limitations with this work, the current study does advance our understanding of sexual identities, child maltreatment, mental health, and substance use using a community sample of emerging adults in Canada. It also highlights the need to further invest in this area so that this work can be examined at the national level with larger nationally representative samples so that all models can be adequately executed. Specifically, recommendations have been put forward for national surveys in Canada to include all child maltreatment types, to ask about experiences in younger children, including younger than 15 years, and to collect longitudinal data (Gonzalez et al., 2021). The findings from the current study further highlight the need for nationally representative data to inform policy and practice and to promote health and well-being at a critical point in development for children, youth, and emerging adults.

Ensuring safe, stable, and nurturing relationships and environments is needed to prevent child maltreatment (Centers for Disease Control & Prevention, 2014). The current research indicates that sexual identity is associated with an increased likelihood of experiencing child maltreatment, depression, anxiety, at-risk alcohol use, and at-risk cannabis use among emerging adults in a community sample. When sexual minority individuals also experience child maltreatment, trends in the data indicate that mental health and substance use outcomes may worsen. More work is needed to confirm these findings using a large nationally representative sample of youth and emerging adults from across Canada. Health and social service professionals working with children and families should be aware of the relationship between sexual identity and poor mental health and substance use outcomes, and how having a child maltreatment history may worsen these associations. It is important that clinicians working with children and families are using evidence-based methods to recognize and safely respond to child maltreatment (Gonzalez et al., 2021; Kimber et al., 2021; Mathews et al., 2022). Preventing violence, improving mental health, and reducing at-risk substance use among sexual minority youth remain public health priorities.

## Contributions to knowledge

What does this study add to the existing knowledge?

- Novel findings are that compared to identifying as heterosexual, sexual identity of homosexual, gay, or lesbian; bisexual; and other identity were associated with an increased likelihood of child maltreatment history, along with an increased likelihood of many poor mental health and substance use outcomes.
- Notably, more significant models were found for depression and anxiety and fewer for at-risk cannabis and at-risk alcohol use among this sample of emerging adults.
- Trends suggest that the association between sexual identity and poor mental health and substance use outcomes may be worsened in some cases if the individual also has a child maltreatment history.

What are the key implications for public health intervention, practice, or policy?

- Promoting and maintaining safe environments for all children is essential to ensure healthy development and to reduce mental disorders and at-risk substance use. This may be especially necessary for children and youth who identify with a sexual identity other than heterosexual, to reduce the occurrence of mental disorders and substance use as they enter adulthood.
- From a societal perspective, it is important to invest in public education and resources that normalize and promote acceptance of all sexual identities and remove any tolerance for violence against children and youth.

## Data Availability

Due to ethics agreements, these data are not allowed to be shared.

## Code availability

N/A.

## References

[CR1] Adamson, S. J., Kay-Lambkin, F. J., Baker, A. L., Lewin, T. J., Thornton, L., Kelly, B. J., & Sellman, J. D. (2010). An improved brief measure of cannabis misuse: The Cannabis Use Disorders Identification Test-Revised (CUDIT-R)☆. *Drug and Alcohol Dependence,**110*(1–2), 137–143. 10.1016/j.drugalcdep.2010.02.017 20347232 10.1016/j.drugalcdep.2010.02.017

[CR2] Afifi, T. O., Mather, A., Boman, J., Fleisher, W., Enns, M. W., MacMillan, H., & Sareen, J. (2011). Childhood adversity and personality disorders: Results from a nationally representative population-based study. *Journal of Psychiatric Research,**45*(6), 814–822. 10.1016/j.jpsychires.2010.11.008 21146190 10.1016/j.jpsychires.2010.11.008

[CR3] Afifi, T. O., Henriksen, C. A., Asmundson, G. J. G., & Sareen, J. (2012a). Childhood maltreatment and substance use disorders among men and women in a nationally representative sample. *The Canadian Journal of Psychiatry,**57*(11), 677–686. 10.1177/070674371205701105 23149283 10.1177/070674371205701105

[CR4] Afifi, T. O., Mota, N. P., Dasiewicz, P., MacMillan, H. L., & Sareen, J. (2012b). Physical punishment and mental disorders: Results from a nationally representative US sample. *Pediatrics,**130*(2), 184–192. 10.1542/peds.2011-2947 22753561 10.1542/peds.2011-2947

[CR5] Afifi, T. O., Mota, N., MacMillan, H. L., & Sareen, J. (2013). Harsh physical punishment in childhood and adult physical health. *Pediatrics,**132*(2), e3335. 10.1542/peds.2012-4021 10.1542/peds.2012-4021 23858428

[CR6] Afifi, T. O., MacMillan, H. L., Boyle, M., Taillieu, T., Cheung, K., & Sareen, J. (2014). Child abuse and mental disorders in Canada. *Canadian Medical Association Journal,**186*(9), E324–E332. 10.1503/cmaj.131792 24756625 10.1503/cmaj.131792 PMC4050024

[CR7] Afifi, T. O., Ford, D., Gershoff, E. T., Merrick, M., Grogan-Kaylor, A., Ports, K. A., MacMillan, H. L., Holden, G. W., Taylor, C. A., Lee, S. J., & Peters Bennett, R. (2017a). Spanking and adult mental health impairment: The case for the designation of spanking as an adverse childhood experience. *Child Abuse and Neglect,**71*, 24–31. 10.1016/j.chiabu.2017.01.014 28126359 10.1016/j.chiabu.2017.01.014 PMC7983058

[CR8] Afifi, T. O., Sareen, J., Fortier, J., Taillieu, T., Turner, S., Cheung, K., & Henriksen, C. A. (2017b). Child maltreatment and eating disorders among men and women in adulthood: Results from a nationally representative United States sample. *International Journal of Eating Disorders,**50*(11), 1281–1296. 10.1002/eat.22783 28990206 10.1002/eat.22783 PMC5698735

[CR9] Afifi, T. O., Salmon, S., Garcés, I., Struck, S., Fortier, J., Taillieu, T., Stewart-Tufescu, A., Asmundson, G. J. G., Sareen, J., & MacMillan, H. L. (2020a). Confirmatory factor analysis of adverse childhood experiences (ACEs) among a community-based sample of parents and adolescents. *BMC Pediatrics,**20*(1), 178. 10.1186/s12887-020-02063-3 32316954 10.1186/s12887-020-02063-3 PMC7171813

[CR10] Afifi, T. O., Taillieu, T., Salmon, S., Davila, I. G., Stewart-Tufescu, A., Fortier, J., Struck, S., Asmundson, G. J. G., Sareen, J., & MacMillan, H. L. (2020b). Adverse childhood experiences (ACEs), peer victimization, and substance use among adolescents. *Child Abuse & Neglect,**106*, 104504. 10.1016/j.chiabu.2020.104504 32402816 10.1016/j.chiabu.2020.104504

[CR11] Anda, R. F., Whitfield, C. L., Felitti, V. J., Chapman, D., Edwards, V. J., Dube, S. R., & Williamson, D. F. (2002). Adverse childhood experiences, alcoholic parents, and later risk of alcoholism and depression. *Psychiatric Services,**53*(8), 1001–1009. 10.1176/appi.ps.53.8.1001 12161676 10.1176/appi.ps.53.8.1001

[CR12] Andersen, J. P., & Blosnich, J. (2013). Disparities in adverse childhood experiences among sexual minority and heterosexual adults: Results from a multi-state probability-based sample. *PLoS ONE,**8*(1), e54691. 10.1371/journal.pone.0054691 23372755 10.1371/journal.pone.0054691 PMC3553068

[CR13] Andresen, J. B., Graugaard, C., Andersson, M., Bahnsen, M. K., & Frisch, M. (2022). Adverse childhood experiences and mental health problems in a nationally representative study of heterosexual, homosexual and bisexual Danes. *World Psychiatry,**21*(3), 427–435. 10.1002/wps.21008 36073708 10.1002/wps.21008 PMC9453895

[CR14] Austin, A., Herrick, H., & Proescholdbell, S. (2016). Adverse childhood experiences related to poor adult health among lesbian, gay, and bisexual individuals. *American Journal of Public Health,**106*(2), 314–320. 10.2105/AJPH.2015.302904 26691127 10.2105/AJPH.2015.302904 PMC4815563

[CR15] Austin, A., Craig, S. L., D’Souza, S., & McInroy, L. B. (2022). Suicidality among transgender youth: Elucidating the role of interpersonal risk factors. *Journal of Interpersonal Violence,**37*(5–6), NP2696–NP2718. 10.1177/0886260520915554 32345113 10.1177/0886260520915554

[CR16] Babor, T. F., Higgins-Biddle, J. C., Saunders, J. B., Monteiro, M. G., World Health Organization. (2001). *AUDIT: The alcohol use disorders identification test: Guidelines for use in primary health care (No. WHO/MSD/MSB/01.6 a)*. World Health Organization.

[CR17] Balsam, K. F., Lehavot, K., Beadnell, B., & Circo, E. (2010). Childhood abuse and mental health indicators among ethnically diverse lesbian, gay, and bisexual adults. *Journal of Consulting and Clinical Psychology,**78*(4), 459–468. 10.1037/a0018661 20658803 10.1037/a0018661 PMC2911995

[CR18] Benedini, K. M., & Fagan, A. A. (2020). From child maltreatment to adolescent substance use: Different pathways for males and females? *Feminist Criminology,**15*(2), 147–173. 10.1177/1557085118810426

[CR19] Bernstein, D. P., & Fink, L. (1998). *Childhood Trauma Questionnaire: A retrospective self-report*. The Psychological Corporation.

[CR20] Centers for Disease Control and Prevention. (2014). *Essentials for childhood: Steps to create safe, stable, and nurturing relationships*. http://www.cdc.gov/violenceprevention/pdf/essentialsforchildhoodframework.pdf

[CR21] Chan, R. C. H., Operario, D., & Mak, W. W. S. (2020). Bisexual individuals are at greater risk of poor mental health than lesbians and gay men: The mediating role of sexual identity stress at multiple levels. *Journal of Affective Disorders,**260*, 292–301. 10.1016/j.jad.2019.09.020 31521866 10.1016/j.jad.2019.09.020

[CR22] Charak, R., Villarreal, L., Schmitz, R. M., Hirai, M., & Ford, J. D. (2019). Patterns of childhood maltreatment and intimate partner violence, emotion dysregulation, and mental health symptoms among lesbian, gay, and bisexual emerging adults: A three-step latent class approach. *Child Abuse & Neglect,**89*, 99–110. 10.1016/j.chiabu.2019.01.007 30654290 10.1016/j.chiabu.2019.01.007

[CR23] Clements-Nolle, K., Lensch, T., Baxa, A., Gay, C., Larson, S., & Yang, W. (2018). Sexual identity, adverse childhood experiences, and suicidal behaviors. *Journal of Adolescent Health,**62*(2), 198–204. 10.1016/j.jadohealth.2017.09.022 10.1016/j.jadohealth.2017.09.022 PMC5803435 29223563

[CR24] Cortés-Tomás, M.-T., Giménez-Costa, J.-A., Motos-Sellés, P., & Sancerni-Beitia, M.-D. (2016). Different versions of the Alcohol Use Disorders Identification Test (AUDIT) as screening instruments for underage binge drinking. *Drug and Alcohol Dependence,**158*, 52–59. 10.1016/j.drugalcdep.2015.10.033 26616473 10.1016/j.drugalcdep.2015.10.033

[CR25] Coulton, S., Alam, M. F., Boniface, S., Deluca, P., Donoghue, K., Gilvarry, E., Kaner, E., Lynch, E., Maconochie, I., McArdle, P., McGovern, R., Newbury-Birch, D., Patton, R., Phillips, C. J., Phillips, T., Rose, H., Russell, I., Strang, J., & Drummond, C. (2019). Opportunistic screening for alcohol use problems in adolescents attending emergency departments: An evaluation of screening tools. *Journal of Public Health,**41*(1), e53–e60. 10.1093/pubmed/fdy049 29590416 10.1093/pubmed/fdy049 PMC6459356

[CR26] Craig, S. L., McInroy, L. B., D’Souza, S. A., Austin, A., McCready, L. T., Eaton, A. D., Shade, L. R., & Wagaman, M. A. (2017). Influence of information and communication technologies on the resilience and coping of sexual and gender minority youth in the United States and Canada (Project #Queery): Mixed methods survey. *JMIR Research Protocols,**6*(9), e189. 10.2196/resprot.8397 28958984 10.2196/resprot.8397 PMC5639209

[CR27] Craig, S. L., Austin, A., Levenson, J., Leung, V. W. Y., Eaton, A. D., & D’Souza, S. A. (2020). Frequencies and patterns of adverse childhood events in LGBTQ+ youth. *Child Abuse & Neglect,**107*, 104623. 10.1016/j.chiabu.2020.104623 32682145 10.1016/j.chiabu.2020.104623

[CR28] Depa, N., Desai, S., Patel, S., Silvi, S., Hanif, S., Rizvi, S., Rahman, F., Ortega, G., Hsieh, Y.-C., Malik, P., Pathrose, R. P. M., Parikh, T., & Mansuri, Z. (2022). Mental health disparities amongst sexual-minority adolescents of the US – A national survey study of YRBSS-CDC. *Psychiatry Research,**314*, 114635. 10.1016/j.psychres.2022.114635 35640323 10.1016/j.psychres.2022.114635

[CR29] Dube, S. R., Anda, R. F., Felitti, V. J., Edwards, V. J., & Croft, J. B. (2002). Adverse childhood experiences and personal alcohol abuse as an adult. *Addictive Behaviors,**27*(5), 713–725. 10.1016/S0306-4603(01)00204-0 12201379 10.1016/s0306-4603(01)00204-0

[CR30] Fairchild, A. J., & MacKinnon, D. P. (2009). A general model for testing mediation and moderation effects. *Prevention Science,**10*(2), 87–99. 10.1007/s11121-008-0109-6 19003535 10.1007/s11121-008-0109-6 PMC2908713

[CR31] Felitti, V. J., Anda, R. F., Nordenberg, D., Williamson, D. F., Spitz, A. M., Edwards, V., Koss, M. P., & Marks, J. S. (1998). Relationship of childhood abuse and household dysfunction to many of the leading causes of death in adults. *American Journal of Preventive Medicine,**14*(4), 245–258. 10.1016/S0749-3797(98)00017-8 9635069 10.1016/s0749-3797(98)00017-8

[CR32] Fortier, J., Taillieu, T., Salmon, S., Stewart-Tufescu, A., Davila, I. G., MacMillan, H. L., Sareen, J., Tonmyr, L., Brownell, M., Nickel, N. C., & Afifi, T. O. (2022). Adverse childhood experiences and other risk factors associated with adolescent and young adult vaping over time: A longitudinal study. *BMC Public Health,**22*(1), 95. 10.1186/s12889-021-12477-y 35027027 10.1186/s12889-021-12477-y PMC8759271

[CR33] Friedman, M. S., Marshal, M. P., Guadamuz, T. E., Wei, C., Wong, C. F., Saewyc, E. M., & Stall, R. (2011). A meta-analysis of disparities in childhood sexual abuse, parental physical abuse, and peer victimization among sexual minority and sexual nonminority individuals. *American Journal of Public Health,**101*(8), 1481–1494. 10.2105/AJPH.2009.190009 21680921 10.2105/AJPH.2009.190009 PMC3134495

[CR34] Gallo, E. A. G., Munhoz, T. N., Loret de Mola, C., & Murray, J. (2018). Gender differences in the effects of childhood maltreatment on adult depression and anxiety: A systematic review and meta-analysis. *Child Abuse & Neglect,**79*, 107–114. 10.1016/j.chiabu.2018.01.003 29428878 10.1016/j.chiabu.2018.01.003

[CR35] Gilbert, R., Widom, C. S., Browne, K., Fergusson, D., Webb, E., & Janson, S. (2009). Burden and consequences of child maltreatment in high-income countries. *Lancet*, *373*(9657), 68–81. 10.1016/S0140-6736(08)61706-7 10.1016/S0140-6736(08)61706-7 19056114

[CR36] GLAAD. (n.d.). *Glossary of terms: LGBTQ*. GLAAD Media Reference Guide – 11th Edition.

[CR37] Gonzalez, A., Afifi, T. O., & Tonmyr, L. (2021). Completing the picture: A proposed framework for child maltreatment surveillance and research in Canada. *Health Promotion and Chronic Disease Prevention in Canada,**41*(11), 392–397. 10.24095/hpcdp.41.11.07 34569775 10.24095/hpcdp.41.11.07 PMC8639173

[CR38] Jiang, J., Tan, Y., & Peng, C. (2023). Sexual orientations in association between childhood maltreatment and depression among undergraduates in mainland of China. *Journal of Affective Disorders,**341*, 194–201. 10.1016/j.jad.2023.08.133 37657619 10.1016/j.jad.2023.08.133

[CR39] Katz-Wise, S. L., Rosario, M., & Tsappis, M. (2016). Lesbian, gay, bisexual, and transgender youth and family acceptance. *Pediatric Clinics of North America,**63*(6), 1011–1025. 10.1016/j.pcl.2016.07.005 27865331 10.1016/j.pcl.2016.07.005 PMC5127283

[CR40] Kimber, M., McTavish, J. R., Vanstone, M., Stewart, D. E., & MacMillan, H. L. (2021). Child maltreatment online education for healthcare and social service providers: Implications for the COVID-19 context and beyond. *Child Abuse & Neglect,**116*, 104743. 10.1016/j.chiabu.2020.104743 32980151 10.1016/j.chiabu.2020.104743 PMC7513691

[CR41] Kingsbury, M., Hammond, N. G., Johnstone, F., & Colman, I. (2022). Suicidality among sexual minority and transgender adolescents: A nationally representative population-based study of youth in Canada. *Canadian Medical Association Journal,**194*(22), E767–E774. 10.1503/cmaj.212054 35667666 10.1503/cmaj.212054 PMC9177208

[CR42] Kokotailo, P. K., Egan, J., Gangnon, R., Brown, D., Mundt, M., & Fleming, M. (2004). Validity of the alcohol use disorders identification test in college students. *Alcoholism Clinical and Experimental Research,**28*(6), 914–920. 10.1097/01.ALC.0000128239.87611.F5 15201634 10.1097/01.alc.0000128239.87611.f5

[CR43] Kroenke, K., Spitzer, R. L., Williams, J. B. W., & Löwe, B. (2010). The Patient Health Questionnaire Somatic, Anxiety, and Depressive Symptom Scales: A systematic review. *General Hospital Psychiatry,**32*(4), 345–359. 10.1016/j.genhosppsych.2010.03.006 20633738 10.1016/j.genhosppsych.2010.03.006

[CR44] Krueger, E. A., Repati, M. L., Harlow, A. F., Unger, J. B., Lee, J. O., Pedersen, E. R., Conn, B. M., Wong, C., Young, L. E., Barrington-Trimis, J. L., & Leventhal, A. M. (2022). Changes in sexual identity and substance use during young adulthood. *Drug and Alcohol Dependence,**241*, 109674. 10.1016/j.drugalcdep.2022.109674 36332590 10.1016/j.drugalcdep.2022.109674 PMC9994583

[CR45] Liskola, J., Haravuori, H., Lindberg, N., Niemelä, S., Karlsson, L., Kiviruusu, O., & Marttunen, M. (2018). AUDIT and AUDIT-C as screening instruments for alcohol problem use in adolescents. *Drug and Alcohol Dependence,**188*, 266–273. 10.1016/j.drugalcdep.2018.04.015 29803033 10.1016/j.drugalcdep.2018.04.015

[CR46] MacMillan, H. L., Fleming, J. E., Streiner, D. L., Lin, E., Boyle, M. H., Jamieson, E., Duku, E. K., Walsh, C. A., Wong, M.Y.-Y., & Beardslee, W. R. (2001). Childhood abuse and lifetime psychopathology in a community sample. *American Journal of Psychiatry,**158*(11), 1878–1883. 10.1176/appi.ajp.158.11.1878 11691695 10.1176/appi.ajp.158.11.1878

[CR47] Manea, L., Gilbody, S., & McMillan, D. (2015). A diagnostic meta-analysis of the Patient Health Questionnaire-9 (PHQ-9) algorithm scoring method as a screen for depression. *General Hospital Psychiatry,**37*(1), 67–75. 10.1016/j.genhosppsych.2014.09.009 25439733 10.1016/j.genhosppsych.2014.09.009

[CR48] Mathews, B., MacMillan, H. L., Meinck, F., Finkelhor, D., Haslam, D., Tonmyr, L., Gonzalez, A., Afifi, T. O., Scott, J. G., Pacella, R. E., Higgins, D. J., Thomas, H., Collin-Vézina, D., & Walsh, K. (2022). The ethics of child maltreatment surveys in relation to participant distress: Implications of social science evidence, ethical guidelines, and law. *Child Abuse & Neglect,**123*, 105424. 10.1016/j.chiabu.2021.105424 34883421 10.1016/j.chiabu.2021.105424

[CR49] McCabe, S. E., Hughes, T. L., Beal, S., Evans-Polce, R. J., Kcomt, L., Engstrom, C., West, B. T., Veliz, P., Leary, K., McCabe, V. V., & Boyd, C. J. (2022). Sexual orientation differences in childhood sexual abuse, suicide attempts, and DSM-5 alcohol, tobacco, other drug use, and mental health disorders in the US. *Child Abuse & Neglect,**123*, 105377. 10.1016/j.chiabu.2021.105377 34773839 10.1016/j.chiabu.2021.105377 PMC9110097

[CR50] Merrick, M. T., Ports, K. A., Ford, D. C., Afifi, T. O., Gershoff, E. T., & Grogan-Kaylor, A. (2017). Unpacking the impact of adverse childhood experiences on adult mental health. *Child Abuse & Neglect,**69*, 10–19. 10.1016/j.chiabu.2017.03.016 28419887 10.1016/j.chiabu.2017.03.016 PMC6007802

[CR51] Morris, J. F., & Balsam, K. F. (2003). Lesbian and bisexual women’s experiences of victimization: Mental health, revictimization, and sexual identity development. *Journal of Lesbian Studies,**7*(4), 67–85. 10.1300/J155v07n04_05 24831385 10.1300/J155v07n04_05

[CR52] Pinto, A. D., Aratangy, T., Abramovich, A., Devotta, K., Nisenbaum, R., Wang, R., & Kiran, T. (2019). Routine collection of sexual orientation and gender identity data: A mixed-methods study. *Canadian Medical Association Journal,**191*(3), E63–E68. 10.1503/cmaj.180839 30665975 10.1503/cmaj.180839 PMC6336479

[CR53] Reichenheim, M. E., Interlenghi, G. S., Ferreira, M. F., & de Moraes, C. L. (2021). The Alcohol Use Disorders Identification Test (AUDIT) in adolescents: Using a model-based approach to identify patterns of alcohol misuse. *Substance Use & Misuse,**56*(13), 1915–1922. 10.1080/10826084.2021.1958859 34396898 10.1080/10826084.2021.1958859

[CR54] Ruiz, A. L., & Font, S. A. (2020). Role of childhood maltreatment on weight and weight-related behaviors in adulthood. *Health Psychology : Official Journal of the Division of Health Psychology, American Psychological Association,**39*(11), 986–996. 10.1037/hea0001027 32969695 10.1037/hea0001027 PMC8381525

[CR55] Salmon, S., Garcés Dávila, I., Taillieu, T. L., Stewart-Tufescu, A., Duncan, L., Fortier, J., Struck, S., Georgiades, K., MacMillan, H. L., Kimber, M., Gonzalez, A., & Afifi, T. O. (2022). Adolescent health outcomes: Associations with child maltreatment and peer victimization. *BMC Public Health,**22*(1), 905. 10.1186/s12889-022-13310-w 35524250 10.1186/s12889-022-13310-w PMC9074223

[CR56] Saunders, J. B., Aasland, O. G., Babor, T. F., De La Fuente, J. R., & Grant, M. (1993). Development of the Alcohol Use Disorders Identification Test (AUDIT): WHO collaborative project on early detection of persons with harmful alcohol consumption-II. *Addiction,**88*(6), 791–804. 10.1111/j.1360-0443.1993.tb02093.x 8329970 10.1111/j.1360-0443.1993.tb02093.x

[CR57] Schnarrs, P. W., Stone, A. L., Salcido, R., Baldwin, A., Georgiou, C., & Nemeroff, C. B. (2019). Differences in adverse childhood experiences (ACEs) and quality of physical and mental health between transgender and cisgender sexual minorities. *Journal of Psychiatric Research,**119*, 1–6. 10.1016/j.jpsychires.2019.09.001 31518909 10.1016/j.jpsychires.2019.09.001 PMC10675992

[CR58] Scott, J. G., Malacova, E., Mathews, B., Haslam, D. M., Pacella, R., Higgins, D. J., Meinck, F., Dunne, M. P., Finkelhor, D., Erskine, H. E., Lawrence, D. M., & Thomas, H. J. (2023). The association between child maltreatment and mental disorders in the Australian Child Maltreatment Study. *The Medical Journal of Australia,**218*(Suppl 6), S26–S33. 10.5694/mja2.51870 37004186 10.5694/mja2.51870 PMC10952950

[CR59] Schultz, N. R., Bassett, D. T., Messina, B. G., & Correia, C. J. (2019). Evaluation of the psychometric properties of the cannabis use disorders identification test - revised among college students. *Addictive Behaviors*, *95* , 11–15. 10.1016/j.addbeh.2019.02.016 30798191

[CR60] Soares, A. L. G., Hammerton, G., Howe, L. D., Rich-Edwards, J., Halligan, S., & Fraser, A. (2020). Sex differences in the association between childhood maltreatment and cardiovascular disease in the UK Biobank. *Heart,**106*(17), 1310–1316. 10.1136/heartjnl-2019-316320 32665362 10.1136/heartjnl-2019-316320 PMC7476280

[CR61] Statistics Canada. (2017). *Winnipeg, CY, Manitoba and Canada (table) released November 29, 2017. *Retrieved February 19, 2025, from https://www12.statcan.gc.ca/census-recensement/2016/dp-pd/prof/details/page.cfm?Lang=E&Geo1=CSD&Code1=4611040&Geo2=PR&Code2=46&SearchText=Winnipeg&SearchType=Begins&SearchPR=01&B1=All&GeoLevel=PR&GeoCode=4611040&TABID=1&type=0

[CR62] Tran, N. M., Henkhaus, L. E., & Gonzales, G. (2022). Adverse childhood experiences and mental distress among US adults by sexual orientation. *JAMA Psychiatry,**79*(4), 377. 10.1001/jamapsychiatry.2022.0001 35195677 10.1001/jamapsychiatry.2022.0001 PMC8867386

[CR63] Tran, N. M., Mann, S., Cortez, M. G., Harrell, B., & Nettuno, L. (2023). Adverse childhood experiences (ACEs) and mental health by gender identity in the United States, 2019–2021. *Preventive Medicine,**175*, 107705. 10.1016/j.ypmed.2023.107705 37722459 10.1016/j.ypmed.2023.107705

[CR64] Walsh, C. A., MacMillan, H. L., Trocmé, N., Jamieson, E., & Boyle, M. H. (2008). Measurement of victimization in adolescence: Development and validation of the Childhood Experiences of Violence Questionnaire. *Child Abuse & Neglect,**32*(11), 1037–1057. 10.1016/j.chiabu.2008.05.003 18992940 10.1016/j.chiabu.2008.05.003

[CR65] Whalen, C. (2022). Article 19: The right to protection from all forms of violence. In Z. Vaghri, J. Zermatten, G. Lansdown, & R. Ruggiero (Eds.), *Monitoring state compliance with the UN Convention on the Rights of the Child. Children’s Well-Being: Indicators and Research* (Vol. 25, pp. 293–302). Springer, Cham. 10.1007/978-3-030-84647-3_30

[CR66] World Health Organization. (2014). *Global status report on violence prevention 2014*.

[CR67] World Health Organization, Butchart, A., Harvey, P., Mian, M., & Fürniss, T. (2006). *Preventing child maltreatment: A guide to taking action and generating evidence / World Health Organization and International Society for Prevention of Child Abuse and Neglect. *World Health Organization*.* https://iris.who.int/handle/10665/43499

